# Multiple enhancer regions govern the transcription of *CCN2* during embryonic development

**DOI:** 10.1007/s12079-017-0440-4

**Published:** 2017-12-18

**Authors:** Stephanie L. Frost, Ke Liu, Ian M. H. Li, Blandine Poulet, Eithne Comerford, Sarah De Val, George Bou-Gharios

**Affiliations:** 1 Institute of Ageing and Chronic Disease, William Henry Duncan Building, University of Liverpool, West Derby Street, Liverpool, L7 8TX UK; 2 Institute of Veterinary Science, Leahurst Campus, University of Liverpool, Chester High Road, Neston, CH64 7TE UK; 3 Ludwig Institute for Cancer Research, Oxford University, Old Road Campus, Research Building, Headington, Oxford, OX3 7DQ UK

**Keywords:** *CCN2*, Development, Enhancer, Transcription

## Abstract

*CCN2* is a critical matricellular protein that is expressed in several cells with major implications in physiology and different pathologies. However, the transcriptional regulation of this gene remains obscure. We used the Encyclopaedia of DNA Elements browser (ENCODE) to visualise the region spanning from 300 kb upstream to the *CCN2* start site in silico in order to identify enhancer regions that regulate transcription of this gene. Selection was based on three criteria associated with enhancer regions: 1) H3K4me1 and H3K27ac histone modifications, 2) DNase I hypersensitivity of chromatin and 3) inter-species conservation. Reporter constructs were created with sequences spanning each of the regions of interest placed upstream of an *Hsp68* silent proximal promoter sequence in order to drive the expression of β-galactosidase transgene. Each of these constructs was subsequently used to create transgenic mice in which reporter gene production was assessed at the E15.5 developmental stage. Four functional enhancers were identified, with each driving distinct, tissue-specific patterns of transgene expression. An enhancer located -100 kb from the *CCN2* transcription start site facilitated expression within vascular tissue. An enhancer -135 kb upstream of *CCN2* drove expression within the articular chondrocytes of synovial joints. The other two enhancers, located at -198 kb and -229 kb, mediated transgene expression within dermal fibroblasts, however the most prevalent activity was found within hypertrophic chondrocytes and periosteal tissue, respectively. These findings suggest that the global expression of *CCN2* during development results from the activity of several tissue-specific enhancer regions in addition to proximal regulatory elements that have previously been demonstrated to drive transcription of the gene during development.

**Electronic supplementary material:**

The online version of this article (10.1007/s12079-017-0440-4) contains supplementary material, which is available to authorized users.

## Introduction

*CCN2*, also known as connective tissue growth factor (CTGF), is an important matricellular protein. As with the other CCN protein family members, *CCN2* has several domains that mediate signalling between cells and the extracellular matrix within a broad range of tissues. Aberrant expression of *CCN2* has been associated with several fibrotic diseases, in addition to being associated with osteoarthritis and cancer (Planque and Perbal 2003; Omoto et al. 2004; Leask et al. 2009).

In vivo manipulation of *CCN2* expression has been a fundamental strategy in understanding the localisation and function of the protein. Perhaps the most profound insight into the role of *CCN2* has come as a result of global knockout of gene expression (Ivkovic et al. 2003). Whilst this is not embryonically lethal, *CCN2* ^−/−^ mice nonetheless exhibit premature mortality caused by respiratory failure as a consequence of rib malformation. This reflects the severe chondrodysplasia demonstrated by these mice. An important aspect of the endochondral-related phenotype exhibited with *CCN2* knockout is defective angiogenesis which reinforces abnormality in the development and maintenance of endochondral growth plate (Ivkovic et al. 2003). This tallies with the presence and function of *CCN2* in endothelial cells and vascular remodelling, which has been well established in several other studies (Shimo et al. 1999; Friedrichsen et al. 2003; Hall-Glenn et al. 2012). Skeletal dysmorphism displayed in *CCN2* ^*−/−*^ mice is compounded by disruption of intramembranous ossification which results in abnormal craniofacial development (Kawaki et al. 2008). As with deletion of the gene, murine skeletal development is also perturbed by overexpression of *CCN2*. When driven by a collagen type XI promoter this causes premature chondrocyte hypertrophy leading to incomplete cartilage development and accelerated angiogenesis; culminating in reduced bone length and density (Nakanishi et al. 2001). This contrasts overexpression directed by a collagen type II promoter which causes increased postnatal bone length and density (Tomita et al. 2013). Further examination of this model has revealed a chondro-protective function of *CCN2* with increased chondrocyte proliferation, supporting findings from the knockout model where chondrocyte proliferation and stress response were impaired (Hall-Glenn et al. 2013; Itoh et al. 2013). These studies therefore indicate that *CCN2* is of imperative importance in the developmental delineation of both cartilaginous and osseous tissues.

Given that transcription is the first step in the expression of a gene, understanding the regulatory sequences through which gene transcription is controlled is therefore critical. A region of 160 kb encompassing -57 kb upstream to +100 kb downstream of the *CCN2* coding sequence (Tg(Ctgf-EGFP)FX156Gsat) has been used to drive the expression of an eGFP reporter gene as a surrogate of endogenous *CCN2* expression (Gong et al. 2003). Studies using this model have described transgene expression within several tissues including retinal vasculature and cartilage (Pi et al. 2011; Hall-Glenn and Lyons 2011). The presence of regulatory sequence within this 160 kb region is reinforced by other studies which have demonstrated the ability of a region spanning from -4 kb to the *CCN2* coding sequence to drive expression of a β-galactosidase reporter gene within the dermal vasculature and intervertebral disc (Huang et al. 2010; Hall-Glenn et al. 2012). The promoter is a non-coding element found within the vicinity of the transcription start site (TSS) for a gene, and serves as the primary point from which basal gene expression is directed and modulated (Roy and Singer 2015)*.* In vitro studies have identified several transcription factors binding motifs within the proximal promoter region for *CCN2*, enabling identification of some signalling pathways that control expression of the gene. Transforming growth factor-beta (TGF-β) was one of the first cell-signalling molecules to be implicated as a regulator of *CCN2* with the discovery of direct interaction through a binding motif within 200 bp from the human sequence start site (Grotendorst et al. 1996). Subsequent studies have demonstrated multiple indirect signalling interactions through which TGF-β can modulate *CCN2* expression including via Smad, MEK/ERK and Ets-1 binding motifs within the promoter region (Holmes et al. 2001; Leask et al. 2003; Geisinger et al. 2012). Several other signalling pathways have been associated with transcriptional regulation of *CCN2* including hypoxia inducible factor-1 (HIF1), thrombin and Sox9 (Chambers et al. 2000; Higgins et al. 2004; Oh et al. 2016). Moreover, there may be competition between transcription factors in binding to shared regulatory sequence motifs in the vicinity of *CCN2*, such as occurs with Sox9 and TCF-LEF (Huang et al. 2010). Thus far, attempts to elucidate the regulatory elements that dictate the transcription of *CCN2* have failed to fully recapitulate the expression pattern of the gene (Friedrichsen et al. 2003; Ivkovic et al. 2003). This therefore suggests that additional non-coding elements located further away from the *CCN2* coding sequence are responsible for wider, cell-lineage specific transcription of the gene*.*

In addition to the promoter, there are several other forms of non-coding regulatory regions; such as enhancers, which confer greater refinement of gene transcription. The purpose of an enhancer is to mediate robust, cell-specific regulation of gene transcription (Heintzman et al. 2009; Kieffer-Kwon et al. 2013). There may be numerous enhancers that regulate the transcription of a single gene, however each may function within a discrete cellular context (Bonn et al. 2012). In addition, the activity of these regions may be seemingly redundant, such as in the case of shadow enhancers where several enhancers drive analogous patterns of target gene expression within a single cell or tissue type (Cannavò et al. 2016). A defining feature of an enhancer is the ability to drive expression independent of its positioning relative to location of the target gene (Nobrega et al. 2003). In terms of mechanistic function, enhancers form dynamic platforms that bring about topological organisation of chromatin that results in the initiation of gene transcription (Spitz and Furlong 2012). Consensus motifs within enhancers are bound by cell lineage-specific transcription factors, triggering a cascade of protein-protein interactions that are prerequisite in gene transcription such as mediator, p300 and cohesin (Petrenko et al. 2016). As this occurs, the chromatin becomes looped and the promoter and enhancer regions are brought within close proximity to one another, whilst the protein machinery required for transcription including RNA polymerase II is assembled at the start site (Kagey et al. 2010; Roy and Singer 2015).

The ability of an enhancer to form these interactions is reliant on permissive nucleosomal structure and epigenetic profile (Thurman et al. 2012). Chromatin state is highly dynamic and specific to cell type and differentiation state, which is reflected in temporospatial precision in enhancer utilisation (Kieffer-Kwon et al. 2013; Nord et al. 2013). Characteristics of chromatin are therefore useful in the identification of enhancer regions and the context in which they may function (Bonn et al. 2012). Firstly, open chromatin is signified by hypersensitivity to DNase I treatment (Thurman et al. 2012). Epigenetic modifications of histone proteins such as acetylation of lysine 27 of histone 3 (H3K27ac) in conjunction with monomethylation of lysine 4 of histone 3 (H3K4me1) are associated with active enhancers. H3K4me1 alone indicates poised enhancers, whereas trimethylation of this residue denotes promoter regions (H3K4me3) (Heintzman et al. 2007; Creyghton et al. 2010; Rada-Iglesias et al. 2011). A further feature of an enhancer is a high degree of sequence conservation between evolutionarily disparate species, denoting selection on the basis of sequence function (Visel et al. 2008). However, sequence divergence does not necessarily deplete an enhancer’s capacity to function (Taher et al. 2011).

The paramount need for rigorous control of *CCN2* expression across many cell and tissue types during development and beyond suggests that it is highly likely that multiple non-coding regulatory elements are utilised in ensuring robust and intricate patterns of physiological gene expression. Identification of these enhancer regions is a fundamental aspect of further understanding the context in which *CCN2* is expressed and the factors that mediate this process. In order to assess this principle, we examined the non-coding region upstream of the *CCN2 gene*, identifying several enhancers that were capable of driving discrete patterns of reporter gene activity in vivo.

## Materials and methods

### Identification of candidate enhancers of ***CCN2***

The Encyclopaedia of DNA Elements (ENCODE) browser (genome.ucsc.edu) build NCBI37/mm9 was used to visualise the region 300 kb upstream of the *CCN2* coding sequence (Waterston et al. 2002; Kent et al. 2002). Given the aforementioned imperative role of *CCN2* in cartilaginous tissue, information pertaining to limb tissue was prioritised in the prediction of enhancer regions. ENCODE assimilated tracks regarding E14.5 limb-specific histone modifications of H3K4me1, H3K3me3, H3K27ac were procured from ENCODE/Ludwig Institute for Cancer Research. DNase I hypersensitivity information was gathered from ENCODE/University of Washington regarding thoracic and pelvic limb bud at E11.5, in addition to lung derived fibroblast at 8 weeks. Information regarding conservation of sequences was gathered from Multiz Alignment (Blanchette et al. 2004). Five candidate enhancers were identified based on convergence of peak locations for each of these tracks. Genomic regions were chosen to encompass the entirety of the widest track peak with additional sequence of +/−100 bp, with each having a final size of approximately 2 kb. This ensured that all potential regulatory sequences were contained within the region, and that insertion into the genome would not perturb function.

### Creation of β-galactosidase reporter constructs

Each of the enhancer regions was amplified from purified from murine genomic DNA (Promega) and cloned into the Hsp68-lacZ-Gateway vector (Pennacchio et al. 2006).

For the −100 kb, −135 kb, −198 kb and -254 kb constructs, regions amplified using PCR with primers; −100 kb F 5’-ACCAGATCAGACACCGAGCAATA, R 5′- TGGTTAATGGCTCACGTGGATTC; −135 kb F 5′- GAAGCGCAAGAAGGAAGACCAAAG, R 5′- CAGCTCCTTTGCCTTTGCACTGTA; 198 kb F 5′- GGTCTTAGGCAAGCAAATCTCTG, R 5′- CATTGAAGAGTCCAAGAAGCAGG. PCR products were cloned into pCR8/GW TOPO entry vector (ThermoFisher). LR recombination reaction was then used to sub-clone enhancer fragment into Hsp68-*lacZ*-Gateway destination vector. For the -229 kb reporter plasmid, the region was amplified with primers containing 5′ sites for ApaI and HindIII restriction enzymes on the forward and reverse primers respectively; F 5′- GTGGTACCGGGCAATTTTAACAAGGCTGAGTA, R 5′- GACGCTAGCTCTCAGGTTCTCAGTCAGTTCTTT. PCR product was agarose gel electrophoresis purified using QIAquick Gel Extraction Kit (Qiagen) before restriction digest, further purification and ligation into Hsp68-*lacZ*-Gateway vector using T4 DNA ligase (New England Biolabs), in accordance with manufacturer’s guidelines. Presence, specificity and orientation of each construct insert was verified using PCR and restriction digest.

### Transgenic animals

All animal work was carried out in compliance with UK Home Office regulation and subject to review from the local institutional ethical committee.

Each of the Hsp68-*LacZ* constructs containing the sequences of interest was linearised via restriction enzyme digest with ApaI and NotI in order to remove bacterial maintenance and selection sequence with agarose gel extraction before and column purification (Merck) and elution in water for embryo transfer (Sigma Aldrich). These constructs were then microinjected into the pro-nuclei of 200 fertilised C57BL6xCBAF1 eggs, and transferred into eight foster mothers as described previously (Chiang et al. 2017). At E15.5, embryos were genotyped using construct specific primers and whole mount embryos were stained as indicated below.

### Staining for β-galactosidase activity

All E15.5 embryos (4–8 pups from each foster mother) were dissected in cold PBS before fixation (0.2% glutaraldehyde, 0.1 M sodium, phosphate buffer pH 7.4, 5 mM EGTA pH 8.0, 2 mM MgCl_2_, and 2% formaldehyde) at room temperature for 45 min Three wash stages of 30 min at room temperature in rinse solution (0.1 M sodium phosphate buffer, 2 mM MgCl_2_, 0.1% sodium deoxycholate, 0.2% NP-40 substitute) were carried out before staining overnight at room temperature in 5-Bromo-4-chloro-3-indolyl-β-D-galactopyranoside (X-gal) stain solution (1 mg/mL X-gal, 5 mM potassium ferricyanate, 4 mM potassium ferrocyanate in rinse solution). The rinse steps were then repeated before whole mount imaging using Olympus SFX10 microscope and overnight fixation in 10% neutral buffered formalin at 4 °C.

For clearing of embryos harbouring the -229 kb enhancer *lacZ* construct, embryos were transferred into 1% potassium hydroxide (KOH) solution at room temperature for 6 days before substitution of solution to 0.8% KOH/20% glycerol and further incubation at room temperature for 6 days. This process was repeated with the glycerol ratio being increased by 20% each time the solution was changed. Upon reaching 100% glycerol, whole mount images were taken of the embryos as before.

### Histology and imaging

Fixed embryos were processed for histology prior to paraffin embedding. Embryos were sectioned (6 μm) in sagittal orientation up to half way into the embryo. Sections were counterstaining with eosin Y and imaged using Olympus BX60 and Carl Zeiss axiocam.

## Results

### The non-coding region upstream of ***CCN2*** contains several enhancers of gene transcription

**Fig. 1:**
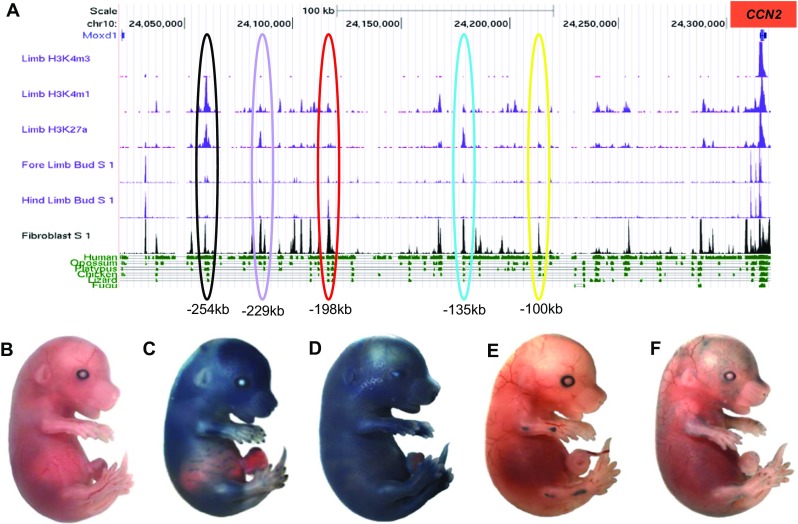
**Multiple enhancers upstream of** ***CCN2*** **function at E15.5**. In silico visualisation of the 300 kb region upstream of *CCN2* using the ENCODE browser (**a**) allowed the identification of candidate enhancers. Regions located -229 kb (**c**), −198 kb (**d**), −135 kb (**e**) and -100 kb (**f**) from the *CCN2* TSS each exhibited capacity to drive *lacZ* reporter gene expression at E15.5. A predicted enhancer located -254 kb from the TSS did not show any transgene activity (**b**)

Five potential enhancers were identified in silico within the murine genome of *CCN2* gene (Fig. 1a). These were located at -254 kb, −229 kb, −198 kb, −135 kb and -100 kb from the TSS. Each of these regions contained track peaks for enhancer associated chromatin characteristics in samples originating from embryonic limb (E11.5 and E14.5). In addition, each demonstrated a high degree of sequence conservation when compared to evolutionarily distant species.

Whole mount imaging of X-gal stained E15.5 embryos allowed gross differences in β-galactosidase transgene activity to be assessed (Figs. 1b-f). For each construct, at least three founders were required to stain in a similar fashion before we report the common features of this enhancer activity. Four of the five predicted enhancer regions; −100 kb, −135 kb, −198 kb and -229 kb, were able to drive transgene activity at this time point using our criteria. Negligible transgene expression was observed in embryos harbouring -254 kb *lacZ* construct across several founders (Fig. 1b). For the -229 kb region superficial transgene expression was observed globally with the exception of the ear, abdomen, paws and tail; although these regions were not completely devoid of staining (Fig. 1c). The strongest transgene expression was observed within skeletal elements, including the parietal and frontal regions of the cranium, ribs and limbs. The region at -198 kb drove the strongest pattern of transgene expression with dermal X-gal staining observed across the whole embryo, with the exception of the digits (Fig. 1d). These patterns contrasted with the staining observed for the -135 kb and -100 kb enhancers which both demonstrated expression within distinct anatomical locations. The -135 kb region drove strong transgene expression in the articular regions of both the fore and hind limbs, with staining restricted to the elbow, wrist, knee and ankle (Fig. 1e). For the -100 kb enhancer, staining was punctate across the dermis (Fig. 1f).

### -100 kb upstream enhancer of *CCN2* functions within dermal microvasculature

**Fig. 2:**
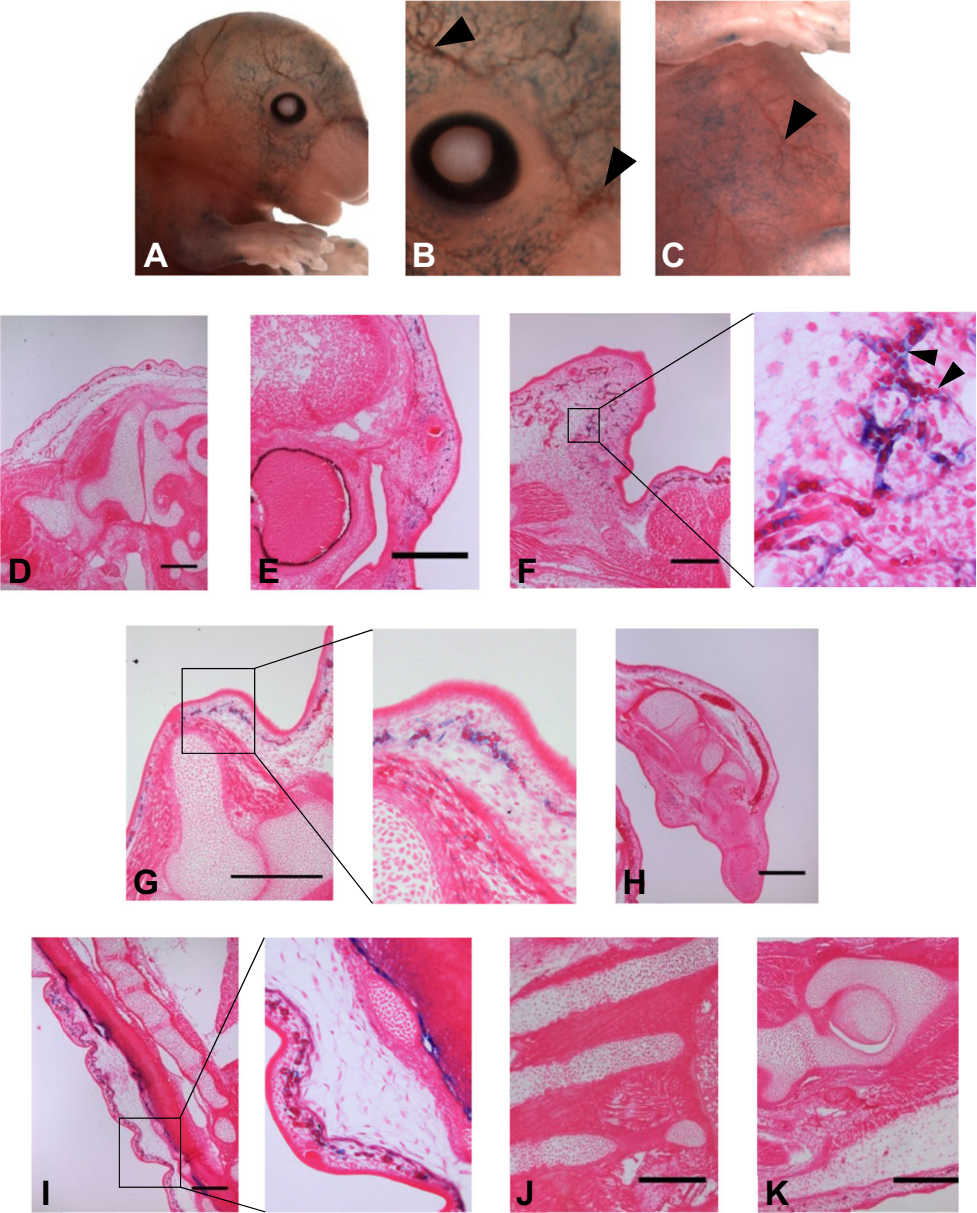
**-100 kb upstream sequence of** ***CCN2*** **gene drives transgene activity within microvasculature.** Larger blood vessels were not stained (arrowheads **b** and **c**) contrasting the punctate staining in the surrounding area. Transgene localisation was concomitant with that of microvasculature and capillary vessel endothelium, with erythrocytes visible within blue branched structure (**f**, black arrowheads). This pattern was observed globally in a highly superficial manner such as in the frontal portion of the craniofacial region (**e** and **f**) and primordial dermis surrounding the fore limb (**g**) and spine (**i**). Staining was not observed within other populations of endothelial cells, such as in the growth plate of endochondral tissue. There was an absence of staining in any other tissue type including cartilaginous tissue of the chondrocranium (**d** and **e**), wrist (**h**), intervertebral disc (**i**), ribs (**j**) and femur (**k**). Early osseous tissue within these regions was also negative for transgene expression (Bar = 100 μm)

Several founder embryos in which β-galactosidase staining was visualised within the vicinity of blood vessels (Fig. 2a-c arrows) were embedded in wax, with histological sectioning revealing that transgene activity only occurred within the microvasculature, and more specifically was located within the superficial vascular plexus of the dermis (Figs. 2f, g and i). β-galactosidase activity was concomitant with the localisation of the developing dermal capillary blood vessels. Furthermore, erythrocytes were localised within areas of staining which also exhibited branching in a vessel like manner (Figs. 2f and g). There was no staining in any other type of blood vessel, nor within cartilaginous, muscle or osseous tissue (Fig. 2a, e, g and h).

### An enhancer located -135 kb upstream of ***CCN2*** facilitates gene expression in chondrocytes

**Fig. 3:**
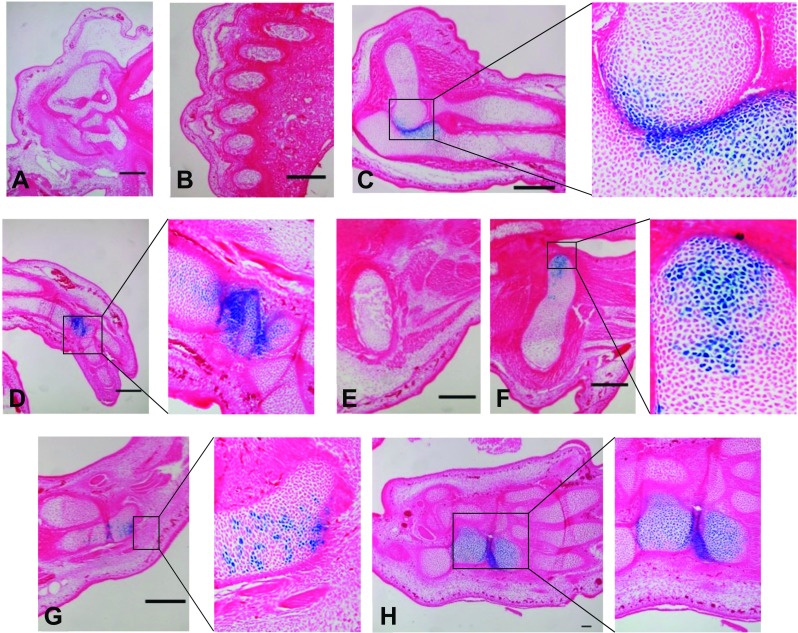
**An enhancer at -135 kb upstream of** ***CCN2*** **drives transgene activity within articular chondrocytes**. Strong transgene expression was observed within the proximity of synovial joints (more specifically the elbow (**c**), wrist (**d**), femoral aspect of the knee (**f**) ankle (**g**) and hind limb paw (**h**)). Hypertrophic chondrocytes, such as in the vicinity of the primary ossification centres of radius and ulna (**c**) in addition to the hip (**e**) and femur (**f**) were not stained. β-galactosidase activity was absent within all other tissues including the chondrocranium (**a**) and ribs (**b**) (Bar = 100 μm)

For the enhancer located -135 kb upstream of the *CCN2* TSS, only cartilaginous tissue exhibited transgene activity (Fig. 3). Moreover, staining was restricted to chondrocyte near the articular joints of the elbow, wrist, knee, ankle and some digits (Figs. 3c, d, f, g and h). There was no transgene activity within other synovial joints including the shoulder and hip. The most striking example of β-galactosidase expression occurred within the elbow where throughout the sagittal plane of the joint, strongly stained chondrocytes were concentrated in the vicinity of the articular surface of the joint, with dissipation of staining towards the growth plate (Fig. 3c). However, the entire population was not positive for transgene activity. There was no X-gal staining within other regions composed of hyaline cartilage; for example within the ribs or cartilaginous anlagen subject to endochondral ossification such as within the forelimb (Fig. 3b and c respectively). In addition, there was no transgene expression within fibrocartilaginous tissue such as within the intervertebral disc. These results therefore indicated that the -135 kb enhancer region was able to drive transgene activity in a highly specific sub-population of articular chondrocytes.

### The -198 kb upstream enhancer of ***CCN2*** demonstrated strong activity in several tissues of mesenchymal origin

**Fig. 4:**
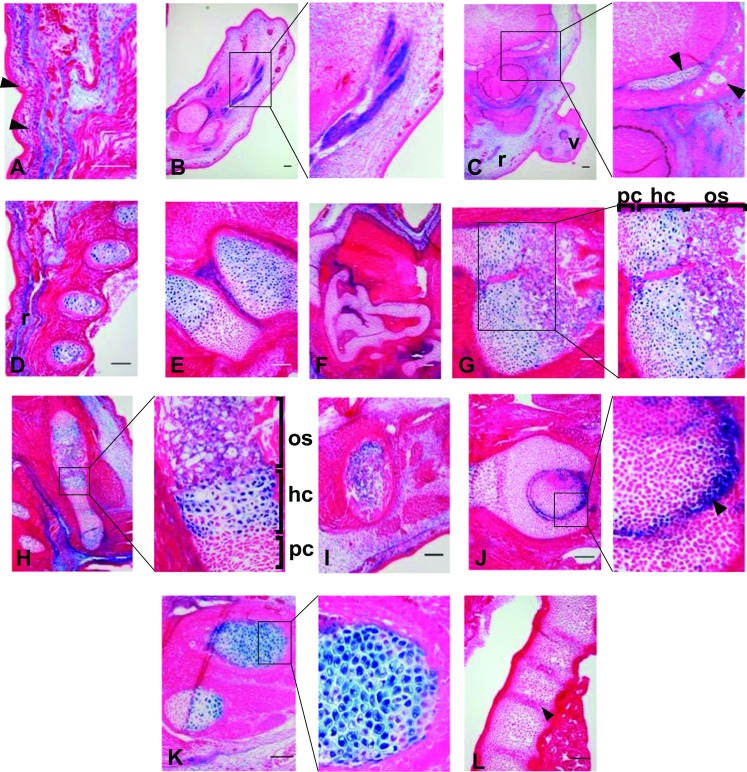
**An enhancer -198 kb upstream of** ***CCN2*** **drives transgene activity within tissue of mesenchymal origin.** Strong transgene expression was observed within dermal mesenchyme globally including within the dorsal (**a** and **d**) and craniofacial (**c**) regions. Further fibroblastic activity was also observed within the tendons of the hind paw (**b**). Hypertrophic chondrocytes stained strongly in a global manner including within the basioccipital bone and atlas (**e**), scapula (**g**), humerus (**h**) and hind limb (**k**). Staining within other types of chondrocytes was limited, with additional activity within articular chondrocytes such as in the hip (**j**, black arrowhead). Punctate staining was observed within the chondrocranium (**c** and **f**) whereas the vertebrae (**l**) and intervertebral disc (black arrowhead) were negative for transgene activity. There was however, some staining within osseous tissue including within the primary ossification centre within the scapula (**h**, os), humerus (**h**, os) and iliac bone (**i**), this was not present in the ossified region of the orbital plate of the frontal bone (**c**, black arrowhead) (Bar = 100 μm)

Histological analysis of β-galactosidase expression driven by the -198 kb enhancer showed expression in several cell lineages. Firstly, fibroblastic cells within the dermis; more specifically within the loosely organised connective tissue of the reticular layer (r), exhibited strong transgene expression (Fig. 4a, c, e). This contrasted with the epidermis, in which there was no transgene expression (Fig. 4a arrowheads). Further transgene expression of fibroblastic origin was also observed within tendinous tissue of the hind paw (Fig. 4b) and the sheath enveloping the vibrissae follicle (Fig. 4c, v). Strong expression of the transgene was also observed in multiple cartilaginous tissues. However, this was not demonstrated in all chondrocytes. Hyaline cartilage displayed prominent X-gal staining, particularly within the ribs (Fig. 4d), basioccipital bone and atlas (Fig. 4e), limbs (Fig. 4h and k) and articular surface of the hip joint (Fig. 4j). However, there was stratification of the intensity of staining within tissue undergoing endochondral ossification which tallied with cellular differentiation state. This was clearly visible in both the scapula and humerus (Fig. 4g and h respectively), where hypertrophic chondrocytes (hc) demonstrated strong transgene activity, contrasting the neighbouring zone of proliferative chondrocytes (pc). The specificity in cartilage sub-type stained was reinforced by a lack of staining of the fibrocartilage within the intervertebral disc (Fig. 4l). We also observed staining of osteoblastic cells within the primary ossification centres of the iliac bone (Fig. 4i), scapula (Fig. 4g os) and long bones including the humerus (Fig. 4h os). There was negligible transgene expression within tissue that undergoes to intramembranous ossification such as within the cranium. For example, the orbital plate of the frontal bone was negative for any staining, whereas the nearby hypochiasmatic cartilage demonstrated punctate X-gal staining (arrowheads Fig. 4c).

### The -229 kb upstream enhancer of ***CCN2*** drives expression primarily within osseous tissue

**Fig. 5:**
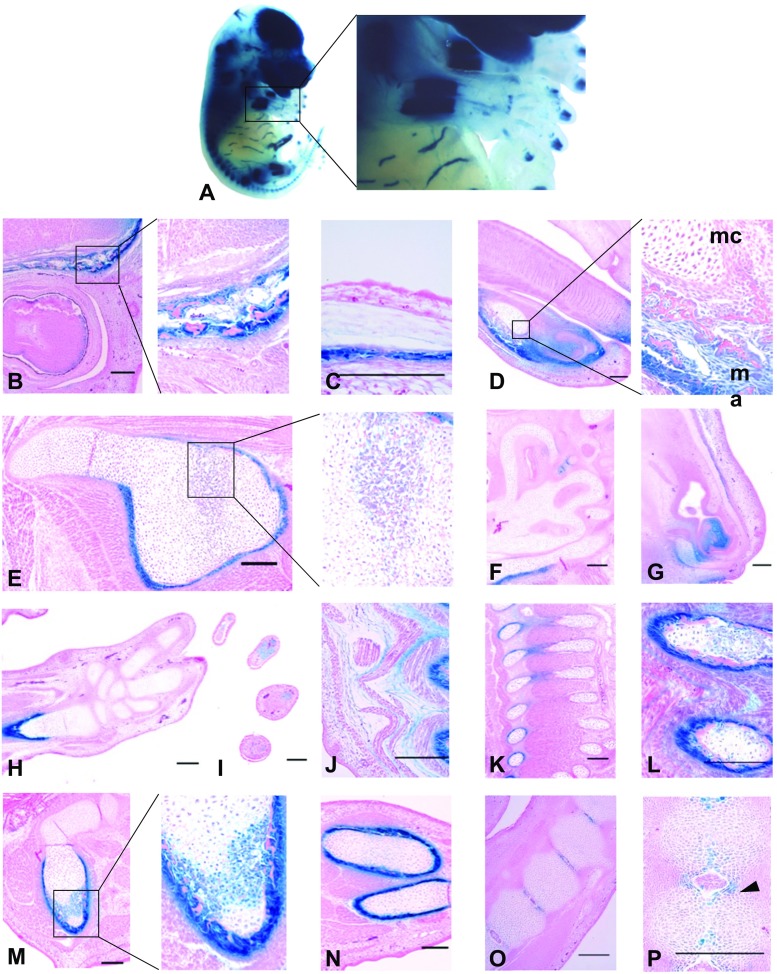
**An enhancer -229 kb upstream of** ***CCN2*** **drives transgene activity within mesenchyme derived tissue.** Whole mount clearing of X-gal stained embryos (**a**) revealed strong staining predominantly within tissue subject to ossification. Marked staining was observed within periosteal tissue within the frontal and parietal bones (**b** and **c**) in addition to the scapula (**e**), ulna (**h**), vertebrae (**k**), ribs (**l**) and hind limb (**m** and **n**). Within the mandibular region (**d**), osseous tissue stained strongly (ma) whereas the Meckel’s cartilage was negative (mc). Staining also occurred within cartilaginous tissue within the chondrocranium (**f**, **g**) and annulus fibrosus of intervertebral disc (**p**, black arrowhead). Transgene activity was also observed within the connective tissue within the dorsal dermis and intercostal regions (**j** and **l** respectively) (Bar = 100 μm)

We conducted soft tissue clearing of embryos exhibiting β-galactosidase expression driven by the enhancer -229 kb upstream of *CCN2* which allowed further refinement of the whole mount imaging (Fig. 5a and 1c respectively). Staining was observed in several tissues derived from mesenchymal origin. As with the -198 kb region, there was staining of the connective tissue within the reticular layer of the dermis (Fig. 5j). However, the most prevalent transgene expression driven by this enhancer occurred within primitive osseous tissue. The prevalence of enhancer activity within this tissue type was exemplified upon examination of the ribcages of cleared whole mount embryos, with the observation that the dorsal areas (which are ossified) stained strongly, whereas the frontal portion that remains cartilaginous did not stain. The process of clearing also enabled the identification of X-gal staining confined to the mid-shaft regions of both fore and hind limbs (Fig. 5a).

Upon sectioning of these tissues it was clear that this staining was specifically within the periosteum and early bone collar. Strong staining consistently occurred around tissue subject to both endochondral and intramembranous ossification, such as in the orbital plate of the frontal bone (Fig. 5b), scapula (Fig. 5e), ribs (Fig. 5k and l) and limbs (Fig. 5h, m and n). However, this pattern of periosteal expression did not extend to all tissues that undergoes ossification. For example, the primitive tissue of the small bones within the paw did not exhibit any transgene activity at this stage of development (Fig. 5h). Transgene expression within cartilaginous tissue was sparse and occurred within the nasal region and fibrocartilage of the intervertebral disc (Figs. 5g, o and p). The small degree of β-galactosidase activity within cartilaginous tissue was exemplified within the mandible, with strong staining of the osseous tissue and no blue staining of the chondrocytes within the Meckel’s cartilage (Fig. 5d ma and mc respectively). Within endochondral tissue, osteoblastic cells within the primary ossification centres of the scapula and proximal portion of the femur exhibited staining, but there was neg ligible staining of the chondrocytic cells throughout the tissue, from the elongated hypertrophic cells proximal to the ossification centre to the densely packed proliferating cells.

## Discussion

Previous studies have established a strong consensus that stringent regulation of *CCN2* expression is fundamental in development and beyond, exemplified by pathology that results when this control is lost (Ivkovic et al. 2003). Thus far, study of the regulation of *CCN2* transcription has focused on the upstream locus in close proximity to the coding sequence, in addition to within the introns and 3′ end of the gene. The aforementioned 160 kb BAC sequence did not demonstrate specific *cis*-acting sequences with capacity to drive tissue specific expression of *CCN2* (Gong et al. 2003). In this study, we investigated the 5′ end of the *CCN2* sequence and have identified four *cis*-acting regulatory regions upstream of the coding sequence within the murine genome. Each of these enhancers was able to drive β-galactosidase reporter gene expression in a tissue-specific manner, primarily within cartilaginous, osseous and vascular cells; partially fulfilling the pattern of endogenous *CCN2* expression at this time-point (Friedrichsen et al. 2003; Ivkovic et al. 2003).

A 2.3 kb sequence located approximately -100 kb from the *CCN2* TSS drove transgene expression within the dermal microvasculature alone, with no activity observed in any other endothelial cell population. This pattern was similar to that of vascular endothelial cadherin, which has previously been shown to be present within developing dermal microvasculature; although this gene is also expressed more widely within the endothelium of soft tissue (Monvoisin et al. 2006). This -100 kb enhancer is therefore utilised to drive *CCN2* expression in a highly cell-specific manner. Understanding of the time-frame in which this enhancer is active was aided by the fact that a smaller fragment of approximately 1.7 kb within this region has been previously screened for enhancer activity as part of the VISTA Enhancer Browser and found to be non-functional at E11.5; enhancer activity is therefore induced between E11.5 and E15.5 (Visel et al. 2007). Microvasculature based reporter gene expression has previously been described within the Tg(Ctgf-eGFP)FX156Gsat model and transgenic mice harbouring a sequence spanning from -4 kb to the *CCN2* TSS (Huang et al. 2010; Hall-Glenn et al. 2012). However, these constructs gave rise to expression within a wider range of vascular elements than the -100 kb enhancer. We speculate that the -100 kb enhancer could provide a compensatory mechanism within the endogenous system, ensuring that disruption of one regulatory sequences function does not result in perturbation of *CCN2* transcription in specific cell type (Perry et al. 2011; Cannavò et al. 2016).

Overlap in transgene expression pattern only accounted for a small proportion of the total activity of the enhancers, with the most substantial transgene activity observed within markedly differing cell types. The overlap in transgene activity occurred within articular chondrocyte cells in the -135 kb and -198 kb enhancers. Both enhancers were active within elbow and knee synovial regions, yet the -198 kb enhancer demonstrated additional function close to the articular surface of hip and shoulder joints. There is some variation in the cellular signalling pathways and transcription factors that dictate the formation of different joints, for example between the knee and elbow, which could account for discretion in the joints in which the enhancers were active (Pazin et al. 2012). Limb and joint development occur in a proximal to distal manner and that there was no staining observed in the stylopod joints of the shoulder and knee for the -135 kb enhancer. We therefore speculate that activity of this enhancer may have been greater in earlier time point; for example at E11.5 or E13.5, with diminishing activity by E15.5.

During embryonic development, the importance of stringent *CCN2* expression within cartilaginous tissue is emphasised by the fact that three of the four enhancer regions identified here; in addition to the previously reported BAC and -4 kb models (Huang et al. 2010; Hall-Glenn and Lyons 2011) exhibited transgene activity in chondrocytes. Moreover, the extent of *CCN2* expression within endochondral cartilage has been associated with differentiation state of the chondrocytes, mainly within the more mature hypertrophic chondrocytes (Friedrichsen et al. 2003; Ivkovic et al. 2003). This pattern is concomitant with that of the transgene expression driven by the -198 kb enhancer where clear stratification of β-galactosidase activity was in line with the differentiation state of the chondrocytes. The proliferative chondrocytes within the resting zone were negative for X-gal staining, whereas the columnar, elongated hypertrophic chondrocytes proximal to the primary ossification centre exhibited strong transgene activity. This suggests perhaps that enhancer activity is directed primarily by transcription factors specific to this stage in endochondral ossification; such as Runx2. We have shown in supplementary Fig. S3 the possible location of this binding site, along with other potential factors that can drive this expression; however more study is required to determine direct interaction. *CCN2* is also expressed by hypertrophic chondrocytes postnatally within secondary ossification centres; further study could also seek to understand whether the -198 kb enhancer functions at this stage or solely during embryonic endochondral ossification (Oka et al. 2007).

The role of *CCN2* in ossification is not limited to the cartilaginous anlagen during endochondral ossification. Indeed, osteoblast proliferation is increased in vitro in the presence of *CCN2* and expression of the gene in osteoblasts has also been observed in vivo during bone growth and remodelling (Safadi et al. 2003). The -229 kb enhancer exhibited the most potent activity within osseous tissue in the primary ossification centres of the scapula and femur. This bone-specific function was most distinguishable in the mandibular tissue, with strong X-gal staining of the osseous tissue and no staining of the chondrocytes within the Meckel’s cartilage; a tissue that endogenously expresses *CCN2* at this time point (Shimo et al. 2004). The strongest utilisation of this enhancer occurred within the periosteal tissue and early cortical bone. Knockout of *CCN2* leads to reduced mineralisation of cortical bone at E15.5, therefore implicating *CCN2* in bone collar formation (Kawaki et al. 2008). This is supported by previous study that has found *CCN2* mRNA localised within periphery of early osseous tissue during embryonic development (Friedrichsen et al. 2003). Moreover, the localisation of this transgene expression tallied with the endogenous patterns of osteoblastic lineage markers Collagen type I and Osterix (Maes et al. 2010).

Although a further mouse candidate enhancer region was located -254 kb from the TSS, this did not drive transcription of the transgene at E15.5 (Fig. 1b). This was surprising given that the -254 kb region contained the largest peaks for enhancer associated traits at E14.5 on the ENCODE browser (Fig. 1a). This finding therefore highlights issues with the assumption that in silico annotation tallies with in vivo function (Dogan et al. 2015).

The specificity in the localisation of transgene expression and therefore utilisation of each of the enhancers described here underlines the importance of these regions in determining niche gene expression during development. Enhancers are increasingly being implicated as key mediators of cell fate determination, which is conferred by plasticity in chromatin state throughout the course of development (Heintzman et al. 2009; Zhu et al. 2013; Huang et al. 2016). *CCN2* has previously been associated with tissues undergoing differentiation and transition of cell type; as occurs in development of cartilage from mesenchymal precursor (Friedrichsen et al. 2003). This lends credence to the notion that the enhancers identified in the current study contribute to cell lineage-determination through stratification of *CCN2* transcription and therefore protein function. However, further study is required to elucidate the specific temporospatial context in which each enhancer functions. For example, assessment of enhancer function within adulthood is a critical aspect of understanding whether function is limited to during embryonic development, or whether it prevails to drive the postnatal transcription of *CCN2*. Furthermore, the enhancers could also function in a latent manner, with environmental stimuli triggering utilisation of an enhancer in order to drive expression of *CCN2* (Nord et al. 2013). The transgene expression patterns observed in the current study are a reflection of the integration of activity from multiple signalling pathways and therefore transcription factors in a cell lineage-specific manner (Kieffer-Kwon et al. 2013; Huang et al. 2016). An important step in future study will be the identification of the key transcription factor consensus binding motifs that underpin the function of each enhancer. For this reason, we have included supplementary Figs. S1-S5 which depict possible binding sites for known transcription factors which have high homology across species that can be used in mutation experiments. This includes predictions of sites within the BAC sequence previously used (S5) which may help identify the shorter elements that drive activity of transgene in vivo. The combination of in vivo study and mutational analysis would represent a powerful tool than assessment of chromatin state in enabling refining understanding of the context in which each enhancer is utilised (Dogan et al. 2015).

**Fig. 6:**
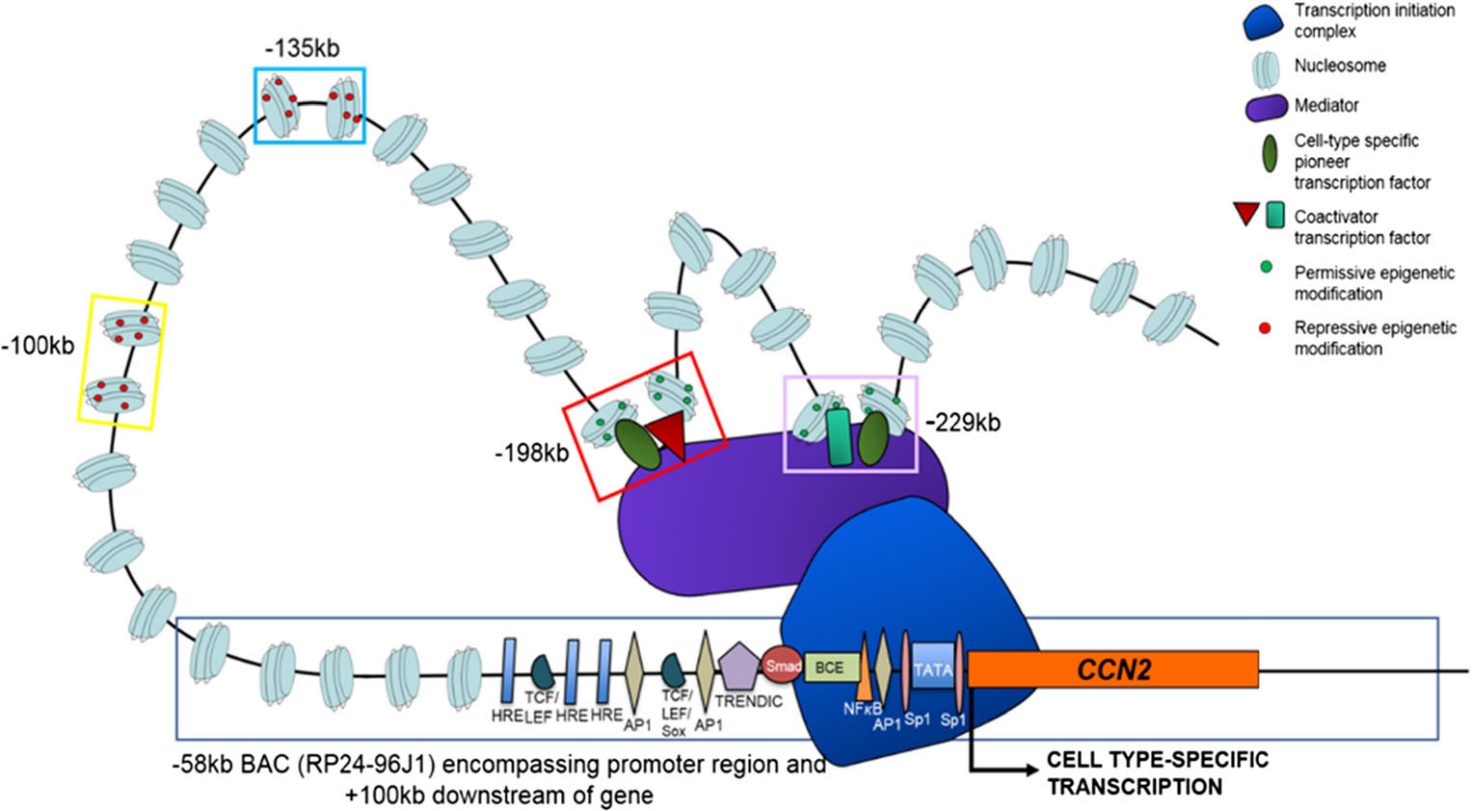
**A potential mechanism through which** ***CCN2*** **transcription is regulated by multiple enhancer regions in a cell type-specific manner.** Open chromatin with permissive epigenetic modification within an enhancer (such as -198 kb and -229 kb) is bound by transcription factors that are cell type-specific, resulting in looping of chromatin. Further protein-protein interaction allows the assembly of the transcription initiation complex at the *CCN2* proximal promoter region, resulting in transcription in a temporospatial manner; such as within dermal fibroblasts at E15.5. The function of other enhancers that may be active at this time-point within other cell-types is prevented by repressive chromatin state (such as with the -100 kb and -135 kb regions). Further regulatory elements within the promoter region and up to -57 kb upstream, as described in previous studies, may also contribute to this refined gene expression

In summary, the expression patterns observed in this study partially recapitulate that of endogenous *CCN2* during embryonic development, indicating that multiple enhancer regions are responsible for the endogenous transcription of *CCN2* (Fig. 6). This therefore suggests that the region of 300 kb upstream of *CCN2* has capacity to function as a topologically associated domain, containing enhancer regions whose composite function facilitates chromatin looping and the induction of *CCN2* transcription within several tissues in a cell-specific manner at E15.5; a potential mechanism for which is shown in Fig. 6 (Dixon et al. 2012). Whilst these findings give an insight into the capacity of non-coding elements to regulate the expression of *CCN2*, further study is required to better characterise these regions and the intricate mechanisms that dictate their function; including knocking out using CRISPR technology. Future examination of enhancer function and sequence could aid in the understanding and amelioration of diseases that involve *CCN2* function as there is a strong consensus that perturbation of topological associated domains and more specifically enhancer activation contributes to pathology (Lupiáñez et al. 2015; Murakawa et al. 2016). Thus, pathological activity of the enhancers described here could contribute to aberrant expression of *CCN2* in disease; for example, in osteoarthritis, especially given the prevalence of chondrocyte based enhancer function.

## Electronic supplementary material

ESM 1 (DOCX 2659 kb)

## Abbreviations

*CCN2*: CCN family member 2
ENCODE: Encyclopaedia of DNA Elements
H3K4me1: Monomethylation of 4th lysine of histone 3
H3K4me3: Trimethylation of 4th lysine of histone 3
H3K27ac: Acetylation of 27th lysine of histone 3
TGF-β: Transforming growth factor beta
TSS: Transcription start site
X-gal: 5-Bromo-4-chloro-3-indolyl-β-D-galactopyranoside
