# Supplementary material for: Multiple enhancer regions govern the transcription of CCN2 during embryonic development
Source: J Cell Commun Signal. 2017 Dec 18;12(1):231–43. doi: 10.1007/s12079-017-0440-4 (PMC5842200; doi:10.1007/s12079-017-0440-4)
Supplement: Supplementary file 1 — (DOCX 2659 kb) [file 12079_2017_440_MOESM1_ESM.docx]

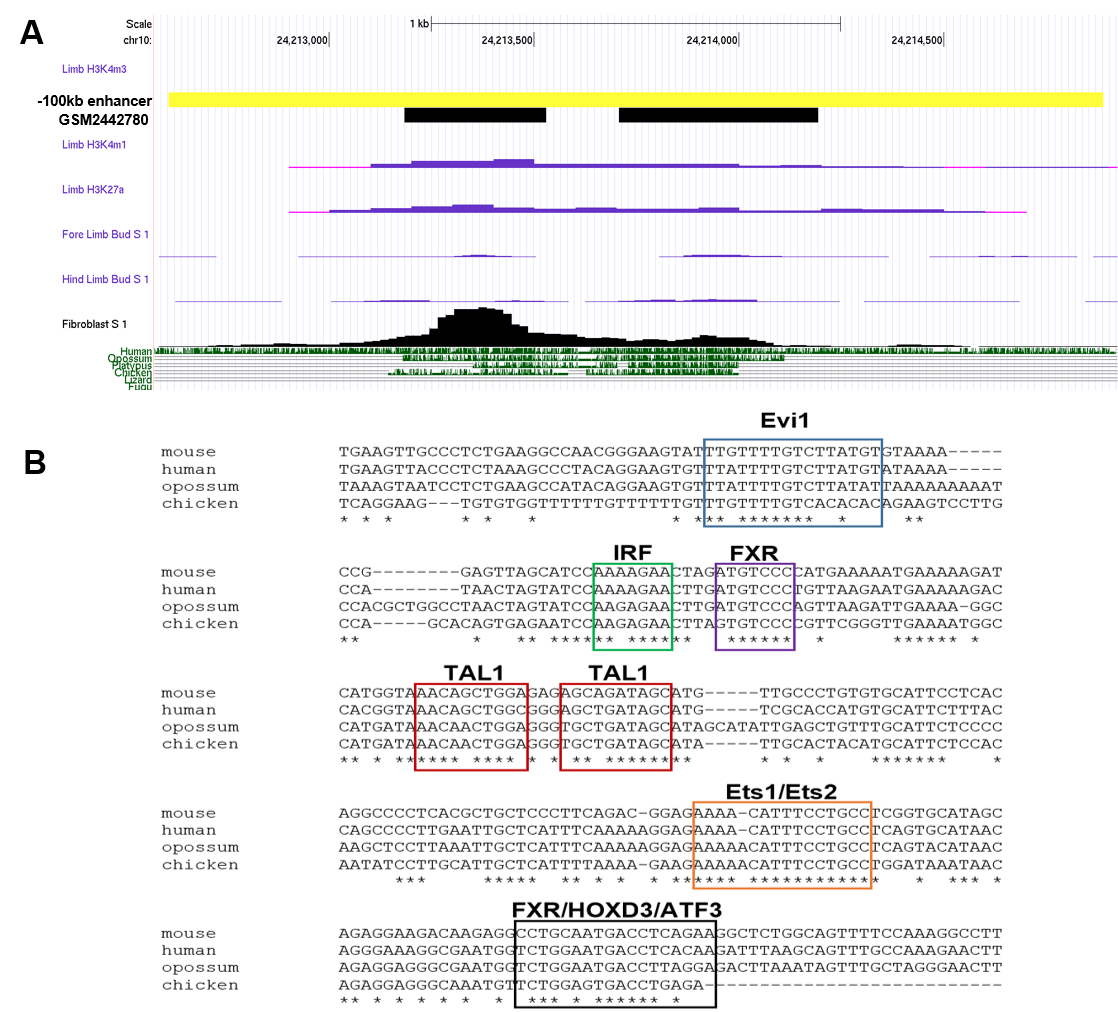


**S1**


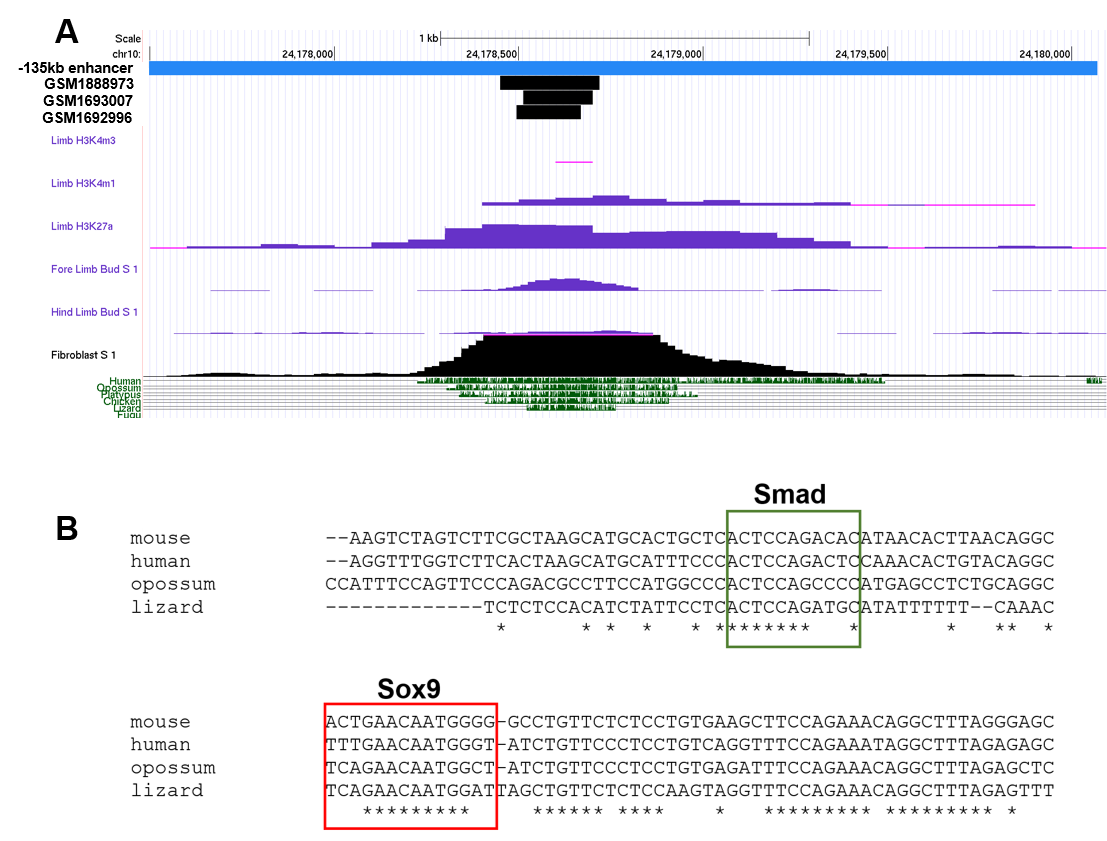


**S2**

**
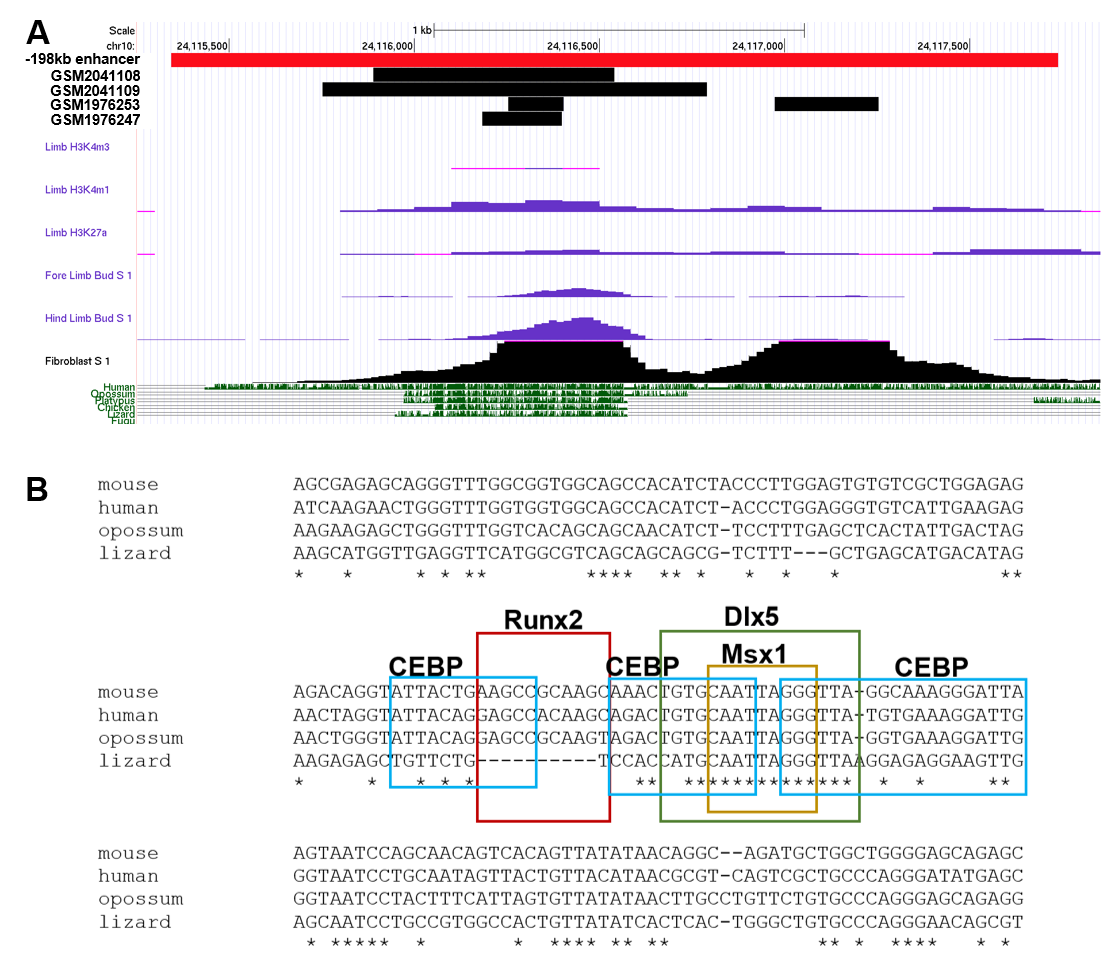
**

**S3**


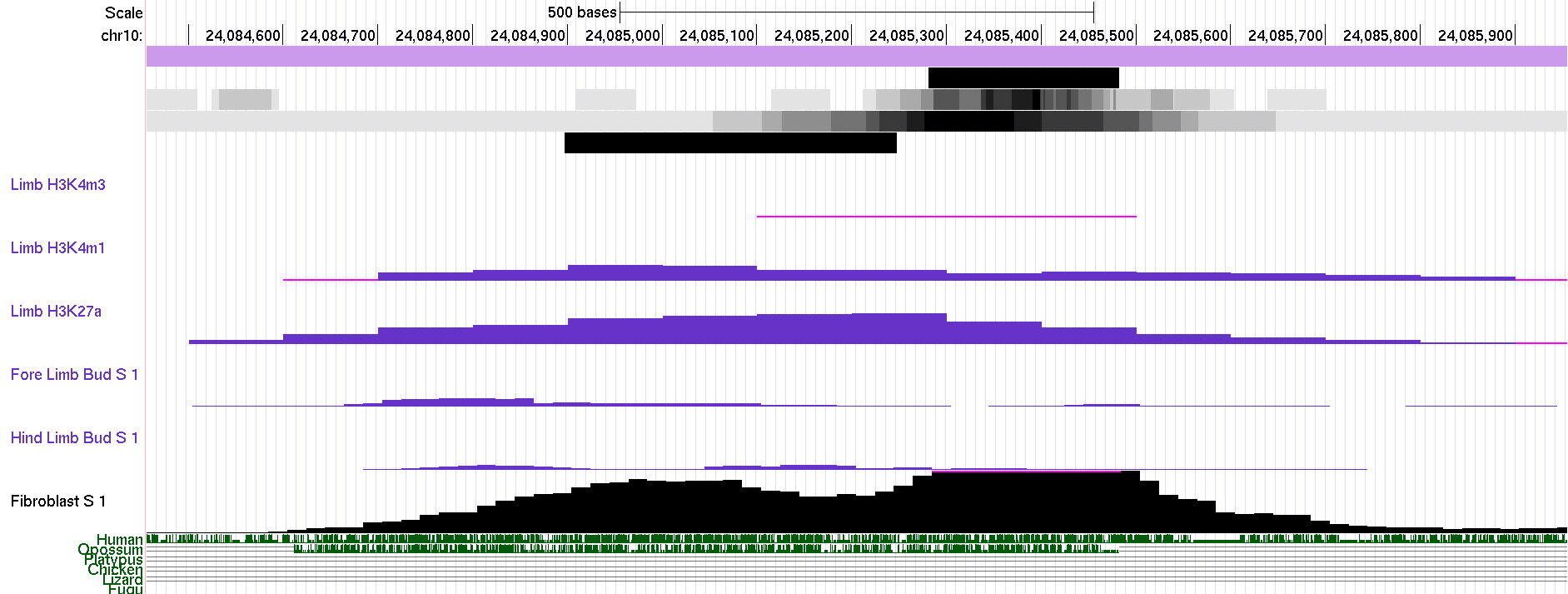


**A**

**-229kb enhancer**

**GSM1027473**

**GSM1027478**

**GSM1027496**

**GSM1888973**


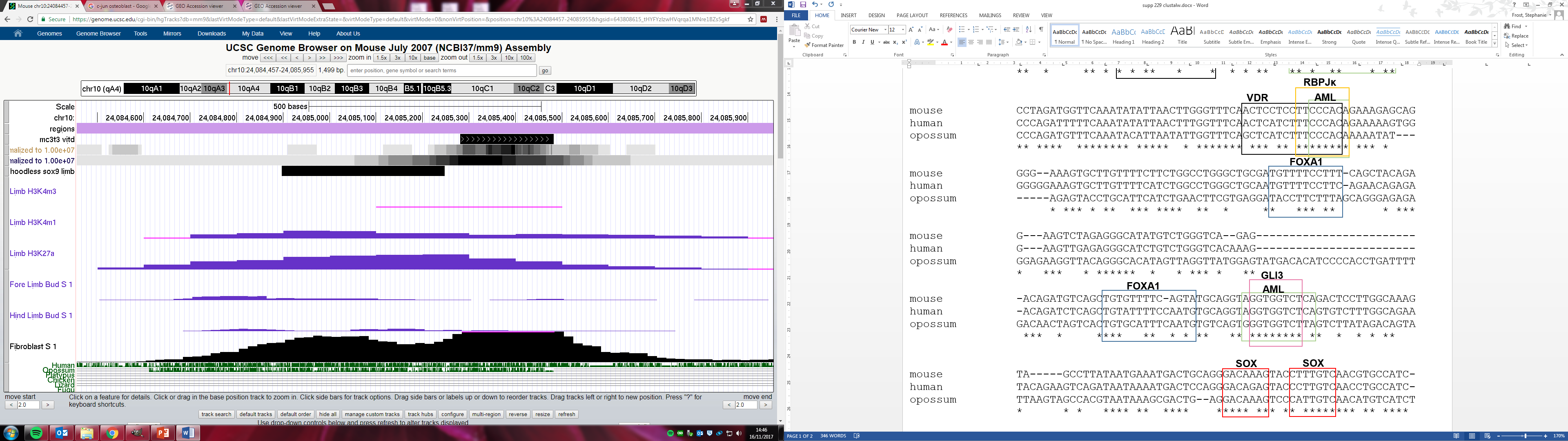


**B**

**S4**


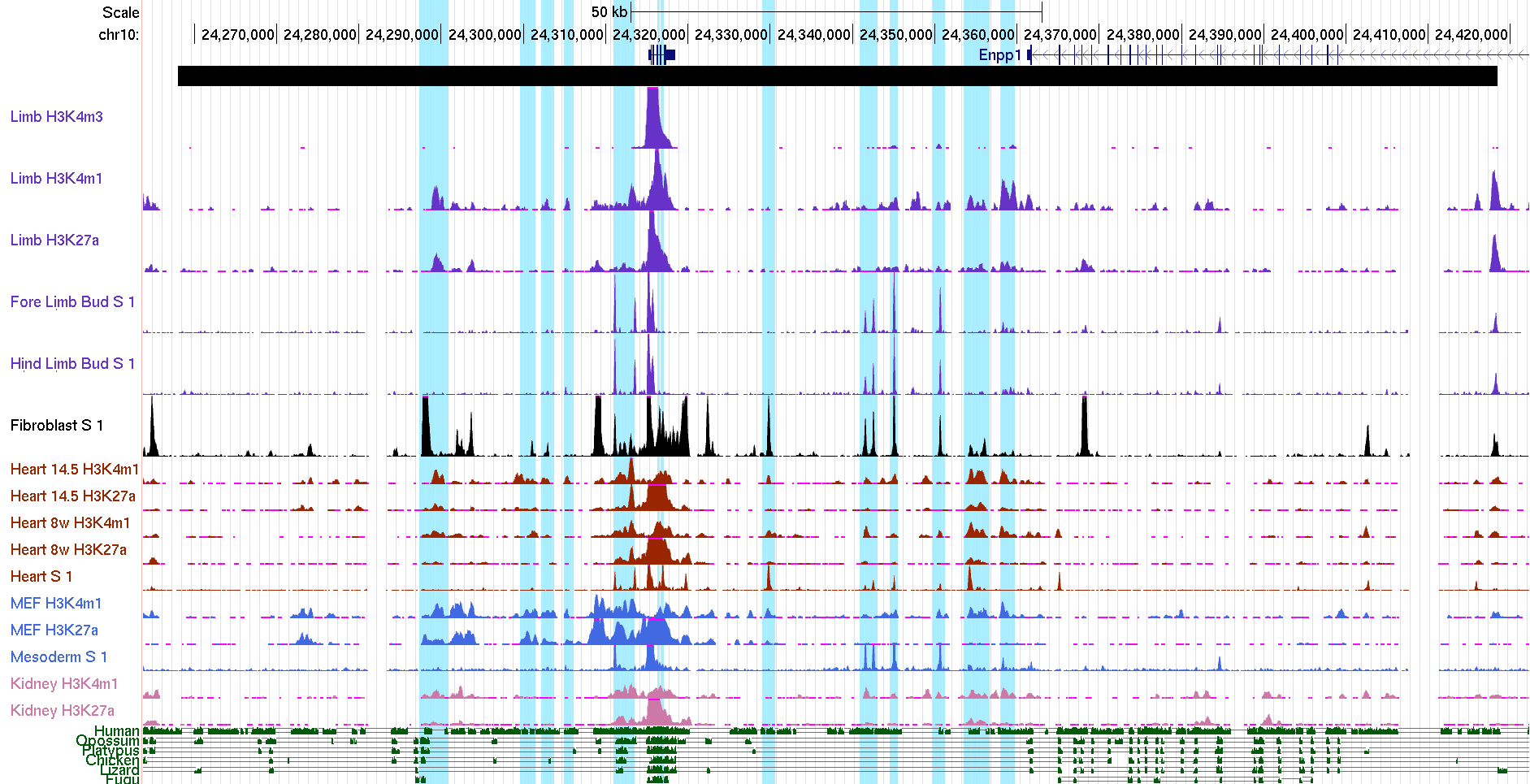


**FX156Gsat (-57kb to +100kb)**

**S5**

**SUPPLEMENTARY FIGURE LEGENDS**

**S1: Potential binding sites for endothelial-lineage related transcription factors within the enhancer located at -100kb upstream of *CCN2*.** ENCODE browser visualisation of the -100kb enhancer with integration of publically available ChIP-Seq data pertaining to Ets-1 binding in HUVEC (Huan *et al* 2016, GEO accession: GSM2442780) which was converted from human hg19 to mouse mm9 genome build (A). Two black bars on track indicate regions where Ets-1 binds to the DNA sequence.

Examination of highly conserved regions of sequence allowed prediction of other consensus motifs for transcription factors that have previously been associated with endothelial cell-lineage stratification (B). The Ets1/Ets2 site (orange box) sits within the 3’ of Ets-1 ChIP-Seq track.

**S2: Prediction of chondrocyte-related transcription factor binding within the -135kb enhancer of *CCN2.*** Given the chondrocyte-specific function of the -135kb enhancer, publically available ChIP-Seq datasets related to master chondrocyte regulator Sox9 capacity to bind DNA within limb tissue at E12 (Garside et al 2015, GEO accession: GSM1888973) in addition to neonatal rib and nasal chondrocytes (Ohba et al 2015, GEO accession: GSM1692996, GSM1693007) were integrated into the ENCODE browser, with visualisation of binding sites within this enhancer region (A). Examination of the sequence where these tracks converged revealed a highly conserved Sox9 consensus motif within this enhancer, in addition to a Smad binding site suggesting that TGF-β may also mediate activity of this enhancer (B).

**S3: Prediction of transcription factors that may facilitate -198kb enhancer activity**

Strong transgene expression within endochondral and osseous tissue driven by the enhancer -198kb is reinforced with the assimilation of publically available ChIP-Seq datasets regarding transcription factors associated with ossification: Runx2 and CBF in SAOS2 osteosarcoma cell line (Shin et al 2016, GEO accession: GSM2041108, GSM2041109), Dlx5 and Sp7 with MC3T3 pre-osteoblast cell line and primary calvarial osteoblasts respectively (Hojo et al 2016, GEO accession: GSM1976253, GSM1976247) (A). Examination of conserved sequence where these peaks converged revealed consensus binding motifs for Runx2 and Dlx5, in addition to further ossification related transcription factors including CEBP and Msx1 (B).

**S4: Prediction of transcription factors that may facilitate function of the enhancer located -229kb upstream of *CCN2****.* Integration of publically available ChIP-Seq datasets allowed visualisation of transcription factors that are capable of binding within the -229kb region (A). These include vitamin D receptor in MC3T3 pre-osteoblast cell line (Meyer et al 2014a, GEO accession: GSM1027473), runx2 in undifferentiated and differentiated MC3T3 cells (Meyer et al 2014b, GEO accessions: GSM1027478 and GSM1027496) in addition to Sox9 (Garside et al 2015, GEO accession: GSM1888973). These transcription factors are predicted to bind; alongside several others, to conserved consensus motifs within this enhancer region (B).

**S5: Potential enhancers that are present in silico within the BAC** FX156G **examined in previous studies.** The region spanning -57kb to +100kb of *CCN2* as utilised in the FX156GSat BAC transgenic construct; including the 4kb previously used by Huang et al (2010) was visualised in the ENCODE browser with assimilation of enhancer associated chromatin attributes (H3K4me1, H3K27ac and S1 DNase I) across several whole tissues in which transgene expression was previously described (Hall-Glenn and Lyons 2011). Blue vertical bands denote predicted enhancer regions where chromatin profile tallies with enhancer function (as shown in our upstream sequences within the results). Peak intensity within these tracks vary with tissue type; suggesting that there could be variation in the regulatory elements utilised between tissue-type.

Enhancers located in the downstream of *CCN2* could well be 5’ enhancers of *Enpp1* rather than 3’ enhancers of *CCN2*.

**SUPPLEMENTARY REFERENCES**

Garside VC, Cullum R, Alder O, et al (2015) SOX9 modulates the expression of key transcription factors required for heart valve development. Development 142:4340–50. doi: 10.1242/dev.125252

Hall-Glenn F, Lyons KM (2011) Roles for CCN2 in normal physiological processes. Cell Mol Life Sci 68:3209–3217. doi: 10.1007/s00018-011-0782-7

Hojo H, Ohba S, He X, et al (2016) Sp7/osterix is restricted to bone-forming vertebrates where it acts as a Dlx co-factor in osteoblast specification. Dev Cell 37:238–253.

Meyer MB, Benkusky NA, Lee C-H, Pike JW (2014a) Genomic determinants of gene regulation by 1,25-dihydroxyvitamin D3 during osteoblast-lineage cell differentiation. J Biol Chem 289:19539–54. doi: 10.1074/jbc.M114.578104

Meyer MB, Benkusky NA, Pike JW (2014b) The RUNX2 cistrome in osteoblasts: Characterization, down-regulation following differentiation, and relationship to gene expression. J Biol Chem 289:16016–16031. doi: 10.1074/jbc.M114.552216

Ohba S, He X, Hojo H, McMahon AP (2015) Distinct Transcriptional Programs Underlie Sox9 Regulation of the Mammalian Chondrocyte. Cell Rep 12:229–243. doi: 10.1016/j.celrep.2015.06.013

Shin MH, He Y, Marrogi E, et al (2016) A RUNX2-Mediated Epigenetic Regulation of the Survival of p53 Defective Cancer Cells. PLOS Genet 12:e1005884. doi: 10.1371/journal.pgen.1005884
